# A Four-Component Framework Toward Patient-Centered, Integrated Mental Healthcare in Kenya

**DOI:** 10.3389/fpubh.2021.756861

**Published:** 2021-12-01

**Authors:** Manasi Kumar, Thomas L. Osborn, Cyrus Mugo, Hossein Akbarialiabad, Osman Warfa, Wangui Muthigani Mbuthia, Christine Wambugu, Carol Ngunu, Fatima Gohar, Shillah Mwaniga, Simon Njuguna, Shekhar Saxena

**Affiliations:** ^1^Department of Psychiatry, University of Nairobi, Nairobi, Kenya; ^2^Department of Psychology, University College London, London, United Kingdom; ^3^Shamiri Institute, Nairobi, Kenya; ^4^Department of Psychology, Harvard University, Cambridge, MA, United States; ^5^Kenyatta National Hospital, Nairobi, Kenya; ^6^Research Center for Psychiatry and Behavioral Sciences, Department of Psychiatry, School of Medicine, Shiraz University of Medical Sciences, Shiraz, Iran; ^7^Student Research Committee, Shiraz School of Medicine, Shiraz University of Medical Sciences, Shiraz, Iran; ^8^Health Service Coordination, Ministry of Health, Nairobi, Kenya; ^9^Universal Health Coverage, Ministry of Health, Nairobi, Kenya; ^10^Adolescent Health Program, Ministry of Health, Nairobi, Kenya; ^11^Preventive and Promotive Health, Nairobi Metropolitan Services, Nairobi, Kenya; ^12^United Nations Children's Fund (UNICEF), ESARO, Nairobi, Kenya; ^13^Adolescents and Key Populations, Nairobi Metropolitan Services, Nairobi, Kenya; ^14^Mental Health and Substance Use Department, Ministry of Health, Nairobi, Kenya; ^15^Department of Global Health and Population, Harvard TC Chan School of Public Health, Harvard University, Cambridge, MA, United States

**Keywords:** mental health, patient-centered care, integrated care, Kenya, universal health care, primary health care

## Abstract

**Background:** How can we fast-track the global agenda of integrated mental healthcare in low- and middle-income countries (LMICs) such as Kenya? This is a question that has become increasingly important for individuals with lived experiences, policymakers, mental health advocates and health care providers at the local and international levels.

**Discussion:** This narrative synthesis and perspective piece encompasses an overview of mental health care competencies, best practices and capacity building needed to fast track patient responsive services. In that vein we also review key policy developments like UHC to make a case for fast-tracking our four-step framework.

**Results:** While there is an increasingly global impetus for integrated mental healthcare, there is a lack of clarity around what patient-responsive mental healthcare services should look like and how to measure and improve provider readiness appropriately. Here, our collaborative team of local and international experts proposes a simple four-step approach to integrating responsive mental healthcare in Kenya. Our recommended framework prioritizes a clear understanding and demonstration of multidimensional skills by the provider. The four steps are (1) *provider sensitization*, (2) *continuous supervision*, (3) *continuous professional training*, and (4) *leadership empowerment*.

**Conclusion:** Our proposed framework can provide pointers to embracing patient-centered and provider empowerment focused quality of care improvements. Though elements of our proposed framework are well-known, it has not been sufficiently intertwined and therefore not been integrated. We think in the current times our integrated framework offers an opportunity to “building back better” mental health for all.

## Background

### Integrated Mental Health Care in the Context of Low- and Middle-Income Countries

Recently, efforts to implement Universal health coverage (UHC) have recognized the need to integrate mental healthcare, a crucial component of UHC, within the broader health services (1–4). These efforts result from the apparent need to incorporate mental healthcare as a core component of health services, which is necessary to actualize UHC (5). Indeed, realizing the urgency and importance of making mental health a crucial component of UHC (6), the WHO recently adopted a Special Initiative for Mental Health with the vision that “all people achieve the highest standard of mental health and well-being” (7). This initiative acknowledged that since mental health problems were responsible for significant personal, communal, and societal suffering, it is impossible to realize the goals of UHC without quality mental healthcare (7). UHC proposes that “all individuals and communities receive the health services they need without suffering financial hardship. It includes the full spectrum of essential, quality health services, from health promotion to prevention, treatment, rehabilitation, and palliative care” (8). Furthermore, UHC must be—by definition—universal; it must cover all populations per the WHO constitution, including individuals with disabilities, refugees, and many other marginalized communities (6, 9–11) which are particularly vulnerable in mental health parlance. UHC is our collective belief that the highest attainable standard of physical and mental health is a fundamental human right and that everyone should have access to quality essential health services without financial constraints. This has in recent years become a critical priority for national governments, and many local and international organizations (8). The prioritization of UHC has come from the broader realization of the United Nations (UN) Sustainable Development Goals (SDGs) with this understanding that poverty reduction, equality and optimal quality of life cannot be attained without realizing this (9).

While the efforts to integrate mental healthcare within the UHC framework have been global, there has been undoubtedly an increased focus on low – and middle–income countries (LMICs) (2, 12–14). This is because LMICs, unlike their higher-income counterparts, have been shown to significantly bear the brunt of mental health problems (15–17) and represent overburdened and poorly invested health systems. These efforts have included attempts at actualizing UHC by international organizations—such as the UN—as well as by national governments and regional health and policy stakeholders. For example, the WHO recently launched a special initiative recognizing the burden of mental health problems in LMICs and commits to raising 60 million dollars. That budget will be used to advance a two-step approach that will involve both the advancement of health policy, advocacy, and human rights and the scaling up of interventions and services across general health settings and other community-based and specialist settings. The aim is to, by 2023, provide access to mental healthcare to 100 million people in 12 LMIC priority countries (7). At the national level, LMIC governments have increasingly made attempts to adopt policy frameworks and build infrastructure to integrate mental health into the UHC agenda. For example, Kenya has recently adopted a mental health policy that aims to set up the policy and infrastructure to advance mental health in the country's UHC agenda with the aim of attaining the highest standard of mental health by 2030 (18).

Regardless of the efforts mentioned above—which should be lauded and are an excellent first step toward integrating mental healthcare within the UHC agenda in LMICs—there is still a concerning need to incorporate mental healthcare as a core component of health services, a crucial and needed step if mental healthcare is to be firmly integrated within the UHC agenda (2, 13, 14, 19, 20). A recent review found that many of the efforts toward integrating mental healthcare in the UHC agenda—and achieving UHC more broadly—have had limited success in many sub-Saharan Africa (SSA) countries (19). This is because most SSA countries face challenges like a more significant proportion of the population living in extreme poverty unable to afford quality healthcare, an informal sector with many uninsured and under-insured people, and a poorly funded primary healthcare system hampering efforts to integrate mental healthcare on ground (19).

The above-mentioned challenges emphasize the need for a dedicated line of work to advance mental healthcare integration within the UHC agenda. Subsequent efforts should be made not only to address health emergencies but also to promote healthier populations in LMICs (21). To do this, three crucial targets should be prioritized with regards to actualizing UHC: (1), *who:* which concerns the inclusion of all people including the poorest and the most vulnerable, (2) *what*: which involves offering a full range of good quality essential services, and (3) *how:* which concerns reducing out of pocket expenses through cost-sharing (e.g., pre-payment and risk pooling). These tangents are reflected in the two broad areas of interventions when it comes to embedding mental healthcare into UHC: strengthening health care systems and workforce capacity to improve accessibility, affordability, and acceptability and expand the availability of services to the neediest.

In the past few years, several frameworks to guide mental healthcare integration within the UHC agenda in LMICs have been proposed. One such framework, by the WHO, postulated several levels at which integrated mental healthcare can be actualized at the grassroots level (6). One highlight of this report was their call for sensible local application of broad care principles, which should address the particular setting/context-relevant nuances while adhering to globally established agreements. This report's important recommendation was that mental healthcare integration should begin at the primary care level because that was the most viable way of closing the mental health treatment gap (6). Though the WHO report conceptualized integration as a cascade of care where different levels interact, they also pointed that integration could be conceptualized in many different ways. Besides the WHO, other financial aid and philanthropic organizations like Bill and Melinda Gates Foundation, World Bank, African Development Bank have also played an essential role in integrated health services while also championing vulnerable populations' health rights and needs (6).

Many local organizations and government agencies also have advanced frameworks to expand UHC in LMICs. For example, the Government of Kenya has through its Vision 2030 Agenda offered a long-term development blueprint that is inspired by a collective aspiration for a better society by the year 2030, which aims to transform the country into a “globally competitive and prosperous country with a high quality of life” (22). Kenya has undertaken efforts to integrate mental healthcare broadly within the healthcare infrastructure. These efforts, conducted by the Ministry of Health and guided by the Kenya Mental Health Policy 2015–2030, have included an increased focus on mental healthcare through policy changes (18, 23). The policy frameworks have suggested action plans designed to improve mental healthcare access and quality care across the country. Similar efforts have also been undertaken at the county level in Kenya that include the recent integrated mental healthcare action plan by the Nairobi County Government for example and several other county mental health plans (24).

The efforts, as mentioned earlier, have been diluted by several factors. One such factor is the lack of progressive and wide-sweeping public policy and practice to sustain this momentum and provide guidance and resources to build these initiatives (2, 4, 13, 19, 20). A primary challenge has been how to connect top-level policy with on-the-ground implementation even where policy exists. Indeed, it is one thing to lay out a plan and a rational course of action that a plan prescribes, but it is another thing altogether to actualize these well-intended plans. This disconnect between policy and practice has resulted in the status quo, a lack of clarity as to how patient responsive mental healthcare should look like at the provider-level, the caregiver team level, and the facility level. One recent article highlighted that in the Kenyan context, this policy-practice disconnect, a lack of clear leadership and communication by government leaders and subsequent detachment by stakeholders at all levels, are the most significant barriers to realizing the UHC agenda (13). Unfortunately, there are many plans, policies, and frameworks, but grassroots-level players do not have the requisite guidance on how to execute these plans. Without clear vision and evaluation plans, it is difficult to measure the extent to which we are making progress toward integrated mental healthcare. For example, it is challenging to measure indices such as provider readiness which is a critical step in assessing integrated mental healthcare action plans.

## Barriers to Integrating Mental Health and Responsive Care

Why is the framework that we propose here needed in the first place? The answer lies in the several systemic barriers that have stood in the way of integrating mental healthcare and responsive care and reducing the disconnect between policy and practice. One recent review, for example, highlighted that even though Kenya had made significant progress toward UHC—the authors used a UHC index and found that Kenya's score on the index had increased from 43.9% in 2003 to 51.6% in 2013—significant barriers such as financial burdens of healthcare still prevented Kenya in its quest for UHC (2). Other barriers such as a lack of democratic prioritization of UHC action plans, differences in prioritizations of action plans by different stakeholders, and a lack of support for important stakeholders—primarily caregivers—have also been also identified (2, 13, 19, 20). The overall UHC agenda is also derailed by lack of a clear financing plan, dysfunctional healthcare system which has been exposed for example during the COVID pandemic with lack of clear coordination between counties and accountability, concerns on supply chains for medical supplies- seen with the challenges in accountability at the Kenya Medical Supplies Authority and frequent healthcare worker strikes and disgruntlement due to low and delayed pay (25). The lack of appropriate monitoring is not limited to mental health. Kenya has a challenge monitoring chronic illnesses and injuries, which limits her responsiveness to the population needs. Chronic conditions, including those related to mental health cause the largest Disability-Adjusted Life-years in LMICs (26).

These barriers can be imagined as occupying different levels. One such level is *patient-level barriers*, the social and cultural barriers that prevent patients from demanding and seeking integrated mental healthcare. This patient-level barrier may include a limited understanding and knowledge of mental health and mental illness—that is often compounded by a societal stigma around mental health help-seeking, patient-level factors such as chronicity of illness, comorbidities, and other adversities often render mental health a low prioritization (for a patient with a chronic infection sometimes mental healthcare can be seen as a luxury) (2, 27, 28). Another set of barriers are *provider-level barriers:* the professional challenges preventing professionals from integrating mental healthcare. This includes, but is not limited to, providers operating in silos, inadequate training and support on mental healthcare, provider stress and burnout, and limited resources (2, 13, 19). Sometimes other provider-level challenges like poor self-care, burnout, and poor mental health may also limit providers' integration of mental healthcare. The overall societal stigma and discrimination against mental health may also affect provider attitudes toward integrating mental healthcare into their services (29). Finally, another level of barriers is the *system-level barriers* that encompass the complex intersectoral policies that hamper implementation (6, 12). These barriers include limited collaboration by policymakers on the ground, an overlap of roles between different players (i.e., national/federal government and state/local governments), which limits responsibility, a lack of system-wide commitment toward integrated mental healthcare, and of course, the complex, and sometimes interpersonal, political dynamics (20). Another interesting system-level barrier is the focus on stigma and discrimination advocacy by policymakers at the expense of, rather than in support of expanding mental healthcare access (6, 12).

## From Responsive, Patient-Centered Towards Integrated Care

An unresponsive health care system would not catalyze the global development agenda including the realization of UHC. For health services to be responsive, health workers need to embed core mental health competencies into their practices. Therefore, it seems that to incorporate UHC policy and practice, a key next step is to introduce responsive care ethos in clinical practice.

If introducing responsive health services is a crucial step toward advancing the UHC agenda in Kenya as in other LMICs. A question that then arises is: *how do we integrate mental healthcare into patient care?* In answering this question, we must acknowledge that mental health programming needs vary for different contexts, including differences in patients, providers, and the facilities (30–34). Undoubtedly, the nature of mental healthcare often depends on a myriad of system level and socio-cultural factors. Unfortunately, this realization has often been construed as an excuse to provide mental healthcare without including mental healthcare experts in the process. Consider task-shifting, of course, the science on task-shifting (sometimes called task-sharing) suggests that lay-providers can effectively and cost-effectively deliver mental health interventions (35–38). But it is incorrect to use task-shifting as the primary, and sometimes only, means of mental healthcare. Can one genuinely claim to do integrated mental healthcare without the inclusion of expert mental health providers or professionals in the formal health delivery process? one of the arguments we make here is that the inclusion of expert mental health providers who can offer specific approaches for age, gender, disability, or cultures and who are also aligned with a patient's overall care is an essential element of integrated mental healthcare. Of course, these professionals can work alongside lay providers, but it must not be construed as a dichotomous either-or decision.

Besides including mental health professionals in clinical care and formal service delivery structures, another crucial element is the privileging of patient perspectives and needs. Patients should not be passive spectators but rather active participants in the caregiving process. This process of integrating patient perspectives and requirements into the service delivery process has been aided by the increasing number of user/patient-centric approaches, including human-centered and design perspectives borrowed from digital health technologies, that have advanced many patient-centered models and privileging of patients' perspectives. Taken together, approaches that involve mental health professionals in the service delivery process that consider and respond to patients' needs and perspectives, are fundamental in integrating mental healthcare into the UHC ethos.

Because of this, we propose a four-step approach that can improve responsive caregiving in Kenya—and other LMICs—and advance the integration of mental health in the UHC agenda. Our proposal is based on the belief that patient-responsive care requires: (a) compassion, empathy, clarity in communication, diligence in action, as well as competence in the specific role and task by the provider at an individual level, (b) good interpersonal communication and teamwork when thinking through care and referral as well as sound follow-up and clarity of roles and responsibilities at the collaborative care or team level, and (c) prompt feedback and communication—including positive support, mentorship and appreciation of the team and individual efforts—(d), follow up on referrals and leading decision-making and management of the team at the facility level. We propose that integrating responsive mental healthcare means using psychological tools to make decisions and empowering teams to be effective, resilient, and responsive. Furthermore, it means using evidence-based mental health interventions to provide care, relief, and psychosocial support to the patient. A framework, such as the one we propose, can help bridge the gap between policy, which in the Kenyan context already exists, and practice.

## Towards a Framework for Integration

In this paper, we advocate for a framework for integration that circumvents some of the aforementioned challenges. our framework in its parts is well-articulated and backed by literature and advocacy however it has not been championed as an integrated, consolidated approach to mental health systems strengthening. We are guided by the belief that integration requires patients' perspectives and inputs to be put front and center, that mental health specialists be engaged in key care decisions, care teams be empowered to be effective, resilient, and responsive, and that evidence-based mental interventions be privileged in providing care, relief, and psychosocial support to patient populations.

**Figure 1:**
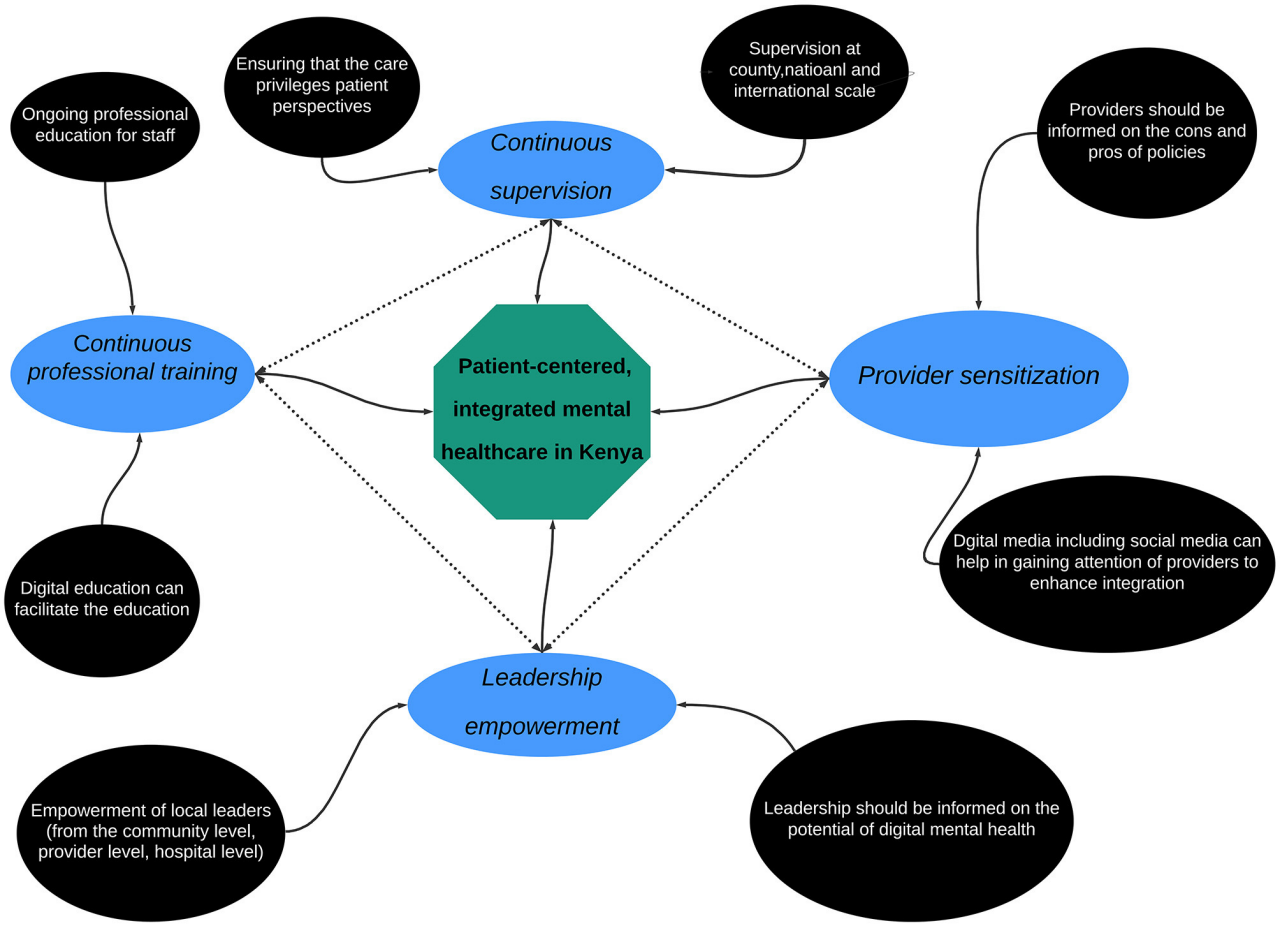
A four-step framework.

How does this framework play out at the individual, team, and provider levels? Our framework calls for empathy, sound communication, diligence in action, and competence in the provider's specific roles and tasks at the individual level. At the care team level, it requires collaborative care that adopts good interpersonal communication, good teamwork in thinking through care and referral and sound follow, and clarity of roles and responsibilities by everyone in the team. Finally, at the facility level, it includes prompt feedback and communication, positive support, mentorship and appreciation of team and individual efforts, follow-up on referrals, and sound management of the team (see Figure 1).

Now we propose concrete on-the-ground steps that catalyze this framework:

1. *Provider sensitization*: One of the reasons for the lack of clarity around the on-the-ground implementation of policy frameworks is that providers are often not adequately sensitized to the nuts and bolts of various policy plans and frameworks which the providers can implement them. Sensitizing providers is a crucial first step in implementing integrated mental healthcare. Suppose providers know that they have to embed mental healthcare in their caregiving approaches and are provided with the tools to do so, as we suggest below; In that case, there will be a lesser disconnect between policy and practice. The current status quo, in which policy frameworks are left to the policymakers' domain, should be abandoned for an approach that emphasizes providers' sensitization on these policy frameworks such as efforts toward WHO QualityRights and Scale up of efforts combining NCDs to mental health, HIV and MNCH. There is precedence and experience from translating policy into practice in HIV care for adolescents, including locally managed training of the adolescent package of services and other HIV-related guidelines (39). Those who have used digital mental health applications to advance the UHC agenda in Kenya have concluded that there is a place for digital technologies because they can facilitate real-time communication, reduce costs, enhance the delivery of evidence-based care by primary health caregivers, promote training, supervision, and continuous support of plans toward the UHC agenda (system level and provider level barriers). Notably, digital mental health is an invaluable source for those avoid mental healthcare services due to stigma, discrimination or racial inequities (patient level barrier). The ongoing COVID-19 pandemic has only accelerated health delivery to Kenya's digital platforms and around the world (40, 41). Taken together, these examples illustrate that policymakers and providers should, to the extent possible, consider the use of digital technologies in mental health services.
2. *Continuous monitoring loops*: Besides sensitization, there needs to be a framework to ensure that integrated mental healthcare that privileges patient perspectives at different caregiving levels is actually and continuously implemented on the ground. This can be done through continuous supervision by local government (i.e., county governments), national government (i.e., Ministry of Health), and even at the international level [i.e., WHO; see (42) for example]. These supervision efforts will help ensure that integrated mental healthcare does not become a one-time initiative but is embedded in health services and heath care institutions. Patient level monitoring helps identify symptoms associated with diseases or disease-driven disorders, which makes it an essential element of psychiatric diagnoses, clinical interventions, and rehabilitation treatments for severe disorders. Incorporating real time data monitoring of the quality of services and patient-provider satisfaction, some through digital solutions, to feed into health service evaluation and creating iterative decision making loops would ultimately strengthen practice based research and quality of care at the same time.
3. C*ontinuous professional training*: Because integrated healthcare requires holistic collaboration between different types of caregivers, there should be avenues for ongoing professional training for all players at all caregiving levels. From nurses and doctors to support staff and policymakers, governments and other entities should provide continued opportunities for professional training and development on integrated mental healthcare and UHC ethos. In 2021, Kenya has a 109% mobile penetration (many have more than one SIM card) and 43% internet penetration (far leading other East African countries) (43), there has been an increased report on the use of digital mental health applications in intervention delivery, training and capacity building, and supervision (44–47). Interventions delivered through digital apps have included the WHO Alcohol, Smoking, and Substance Involvement Screening Test (ASSIST) and its linked brief intervention (BI) (44), brief single-session interventions for adolescent mental health (45), as well as the mobile-based mental health Global Action Global Action Programme Intervention Guide (mhGAP-IG) (48)—which was designed by WHO to help staff diagnose and manage high priority mental health issues in low resource environments (48). Digital trainings can enable the agenda of continuous professional development by offering alternative, enriched and flexible modalities that can be leveraged to enhance learning and supportive mentorship. It proves to be time efficacious as well when health care workers are offered alternatives to in-person trainings.
4. *Leadership empowerment*: As is often the case, the extent to which initiatives succeed often depends on the extent to which the leadership is empowered to succeed. This is definitely the case with UHC. Because mental health problems and their treatments differ from culture to culture, context to context, it has been suggested by some observers that the empowerment of local leaders (from the community level, provider level, hospital level) is an essential step for the success of mental healthcare delivery. At every level, the leadership should be empowered to be able to make and execute context-specific decisions. To facilitate and enhance the use of digital mental health applications in implementing our proposed four-step approach and advancing the UHC agenda in Kenya, we recommend that policymakers and providers in Kenya consider the following considerations leveraging digital tools. (a) Developing proper legislation concerning digital mental health issues, especially around the issues of privacy, inclusivity, data access, sharing, security, and operability. With stringent health governance and stewardship planning, the Ministry of health should promote national intersectoral collaborations to leverage the capabilities of other stakeholders. Collaboration with international organizations such as WHO, UNICEF or other LMICs and best practices to integrate digital mental health in their PHCs successfully. (b) Designing educational platforms and curriculum for digital mental workers to train educators at both advanced and primary levels. The Ministry of Health should work toward increasing digital health literacy among the general population, who are the receivers of such services. Because of the progressive nature of this field, there should be regular sessions aimed at revising the digital health policies to diminish adverse effects and counteract threats.

## Some Exemplars of Integration

We will now illustrate how this framework can be implemented by highlighting recent efforts—at the national and county level—in Kenya. At the national level, the Ministry of Health (MOH) in Kenya (as part of Kenya Health Policy 2014–2030 aimed toward attaining the highest standard of health) has recently adopted the Kenya Mental Health Policy 2015–2030. One reason for this framework is the need to integrate mental health services with essential health programs in Kenya (known as the Kenya Essential Package for Health) and align the mental health agenda in Kenya with national and international efforts. The framework aims to address five key barriers, including (1) a lack of integration of mental health with primary care, (2) inadequate human resources for mental health, and (3) a lack of public mental health leadership. There are three key strategies that the MOH is presently actively pursuing to achieve these objectives. The first is integrating mental health in the Health Information System (HIS); HIS is the electronic medical records system used in Kenya. These efforts will promote the embedding of mental healthcare into primary care. The second is the continued investment in mental health through providing infrastructure and financing for leadership development, human resource capacity building, and providing health products and technologies that help further integrate mental healthcare into primary care. Finally, the MOH has developed and implemented an evaluation system to ensure that there is continued supervision and support at all caregiving levels.

Apart from these efforts at the national level, there have also been recent efforts at the county level to promote UHC. These efforts include those of Nairobi County, which through its County Integrated Development Plan (CIDP), seeks to integrate mental healthcare through its key objective of “halting and reversing the rising burden on non-communicable diseases.” In the CIDP, the County government aims to achieve this objective through strategies such as improving the efficiency of service delivery, comprehensive leadership and governance on the health agenda, equitable distribution of human resources, and promoting universal access to essential health products and technologies. Further, the county is creating an enabling legal framework for the UHC agenda, mobilizing and appropriating adequate resources for the agenda's implementation, and providing training, support, and oversight at various caregiving levels. While it is still early days to know if these efforts will be successful, we believe that as they combine provider sensitization, continuous supervision, professional training, and leadership empowerment, they are a promising step in the right direction.

The “how-to” gap is still enormous in mental health integration in the context of ongoing UHC mobilization. We want to underscore that each of these components gets studied and implemented on its own however our recommendation is to introduce these components at the same time altogether. We think that leveraging digital solutions offer an opportunity to speedily implement and test the impact of these components in real time. It has been suggested that monitoring universal health coverage (UHC) focuses on information on health intervention coverage and financial protection in general but this is also true for mental health field. Investment in provider sensitization, monitoring loops, their capacity building and leadership development is critical toward developing a mental health friendly system and service structure.

## Summary and Conclusion

Here, we have shown a need to accelerate the efforts for integrated mental healthcare in LMIC contexts such as Kenya. We have highlighted worthwhile efforts aimed at this and pointed out that these efforts are weakened by a lack of clarity into how on-the-ground implementation should look like. To address this concern, we have proposed a four-component approach that can reduce the disconnect between policy and practice.

## Data Availability Statement

The original contributions presented in the study are included in the article/supplementary material, further inquiries can be directed to the corresponding author/s.

## Author Contributions

MK: conception of the study, first draft generation and revisions of the paper, approval of the final draft, and overall oversight. TO, CM, and HA: revision of drafts, literature review and editing based on feedback from experts, and quality control. OW, WM, CW, CN, SS, SM, FG, and SN: manuscript revision, expert feedback from their fields of specializations, and approval of the final draft. All authors contributed to the article and approved the submitted version.

## Funding

Research reported in this publication was supported by the Fogarty International Center of the National Institutes of Health under Award Number K43TW010716, which supported the contributions of MK to this work.

## Author Disclaimer

The content is solely the responsibility of the authors and does not necessarily represent the official views of the National Institutes of Health.

## Conflict of Interest

The authors declare that the research was conducted in the absence of any commercial or financial relationships that could be construed as a potential conflict of interest.

## Publisher's Note

All claims expressed in this article are solely those of the authors and do not necessarily represent those of their affiliated organizations, or those of the publisher, the editors and the reviewers. Any product that may be evaluated in this article, or claim that may be made by its manufacturer, is not guaranteed or endorsed by the publisher.
